# Artemisinins as Anticancer Drugs: Novel Therapeutic Approaches, Molecular Mechanisms, and Clinical Trials

**DOI:** 10.3389/fphar.2020.529881

**Published:** 2020-10-06

**Authors:** Cangcang Xu, Huihui Zhang, Lingli Mu, Xiaoping Yang

**Affiliations:** Key Laboratory of Study and Discovery of Small Targeted Molecules of Hunan Province, Department of Pharmacy, School of Medicine, Hunan Normal University, Changsha, China

**Keywords:** artemisinins, antitumor, mechanism of action, clinical trials, therapeutic approach

## Abstract

Artemisinin and its derivatives have shown broad-spectrum antitumor activities *in vitro* and *in vivo*. Furthermore, outcomes from a limited number of clinical trials provide encouraging evidence for their excellent antitumor activities. However, some problems such as poor solubility, toxicity and controversial mechanisms of action hamper their use as effective antitumor agents in the clinic. In order to accelerate the use of ARTs in the clinic, researchers have recently developed novel therapeutic approaches including developing novel derivatives, manufacturing novel nano-formulations, and combining ARTs with other drugs for cancer therapy. The related mechanisms of action were explored. This review describes ARTs used to induce non-apoptotic cell death containing oncosis, autophagy, and ferroptosis. Moreover, it highlights the ARTs-caused effects on cancer metabolism, immunosuppression and cancer stem cells and discusses clinical trials of ARTs used to treat cancer. The review provides additional insight into the molecular mechanism of action of ARTs and their considerable clinical potential.

## Introduction

Cancer is becoming a severe health problem internationally and is one of the most deadly diseases (Siegel et al., 2016). Conventional cancer therapy, especially chemotherapy, provides limited efficacy by DNA damage (Li et al., 2017), but has the problems of formidable side effects and drug resistance. To discover novel potent and safe chemotherapeutic agents or seek better curative methods, medicinal chemists, pharmaceutists and pharmacologists have completed many investigations.

**Figure 1:**
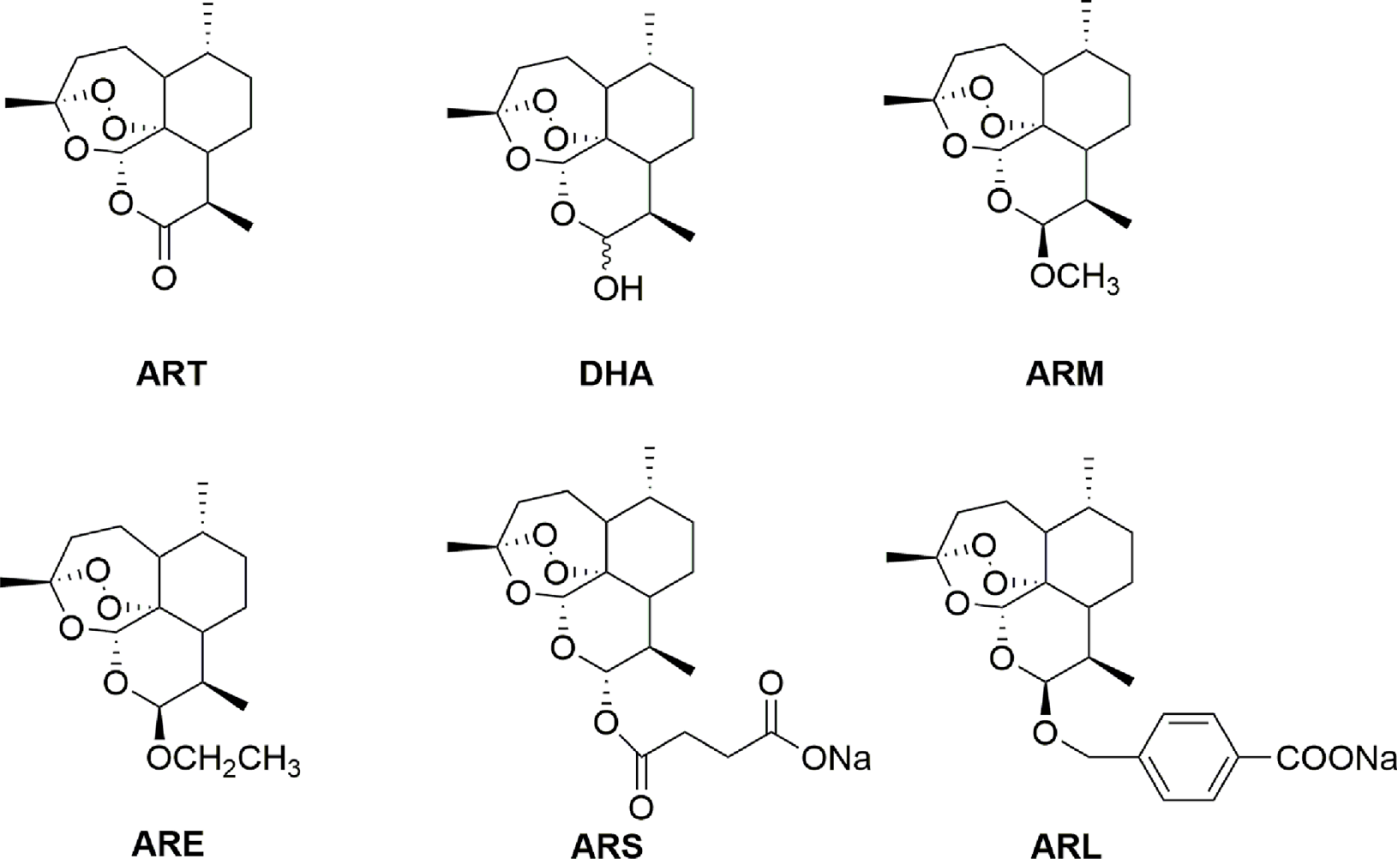
Artemisinin and its derivatives.

Artemisinin (ART), bearing a peroxide bridge in its sesquiterpene lactone structure, was extracted and separated from *Artemisia annua* L. (sweet wormwood), which has been used for treatment of fevers and chills as one of the famous Chinese traditional medicines for thousands of years (Klayman, 1985; Li and Wu, 2003; Cui and Su, 2009; Tu, 2016). ART can be reduced to dihydroartemisinin (DHA) by using sodium borohydride in high yields. Thus, this compound was used as a starting material to prepare the first-generation derivatives including artemether (ARM) and arteether (ARE), and sodium artesunate (ARS) as well as sodium artelinate (ARL) collectively termed as artemisinins (ARTs) (**Figure 1**). As first-line antimalarial medicines, ARTs are safe, low-toxic and well tolerable. However, the neurotoxicity has raised concerns about the safety of ARTs. One of them, ARL was withdrawn from further drug development program because of higher neurotoxicity (Li et al., 2005; Xie et al., 2005). Recently, extensive anticancer effects of ARTs were reported (Ho et al., 2014; Liu X. et al., 2019). More importantly, ARTs have been attractive cancer therapeutic drug candidates with high selectivity, while the neurotoxicity occurred inescapably in clinical studies of cancer at high dosage (Deeken et al., 2018; Von Hagens et al., 2019). Furthermore, the poor solubility of ARTs as well as a short half-life and low bioavailability upon oral administration lead to urgent needs for the finding of novel chemical structures to solve these problems (Crespo-Ortiz and Wei, 2012; Li et al., 2016). To overcome these drawbacks, some novel chemical structures of ARTs have been developed as cancer candidates including mitochondria-targeted derivatives and hybrids while new therapeutic approaches of novel nano-formulations and combination therapy were explored as well (Zhang X. et al., 2015; Zhang et al., 2016; Gao et al., 2018; Suraweera et al., 2018; Hu et al., 2019; Nosrati et al., 2019; Wang Y. et al., 2019; Fröhlich et al., 2020; Zhang et al., 2020).

In addition, the mechanisms of antitumor action are investigated but incompletely elucidated. The mechanism of antitumor action of ARTs mainly involved in apoptotic cell death which has been confirmed by most literatures. Recognized endoperoxide bridge pharmacophore could be reduced by heme or free ferrous iron to generate carbon free radical and reactive oxygen species (ROS) (Firestone and Sundar, 2009; Stockwin et al., 2009; Greenshields et al., 2019). Excess production of ROS is known to cause apoptotic cell death. However, new mechanisms of action in antitumor activity of ARTs by affecting non-apoptotic cell death including oncosis, autophagy and ferroptosis were also found (Du et al., 2010; Zhou et al., 2013; Ooko et al., 2015; Shi et al., 2017; Jiang et al., 2018). Other multiple hallmark events of cancer development and progression were also affected by ARTs including the suppression of cancer cell proliferation, anti-angiogenesis, anti-cancer metastasis and invasion, induction of cell cycle arrest, disruption of cancer signaling pathway and regulation of tumor microenvironment (Efferth, 2017b; Zhang Y. et al., 2018). In tumor microenvironment, there are four types of cells that inhibit immune function including regulatory T cells (Tregs), myeloid-derived suppressor cells (MDSCs), tumor-associated macrophages and cancer-associated fibroblasts. As a result, tumor-specific T cells are unable to enter tumor tissues or their functions are impaired after entering the tissues. This indicates that inhibiting immunosuppression will be beneficial for cancer therapy. Furthermore, it is well-known that cancer cells are different from normal cells with rapid proliferation and metabolic changes especially glycolytic metabolism. Fortunately, ARTs showed antitumor activity by affecting immunosuppression and cancer metabolism (Zhang et al., 2014; Cui et al., 2015; Li S. et al., 2019; Zhu et al., 2019; Gao et al., 2020). The pace on the study of mechanisms of action of ARTs still doesn’t stop after receiving these exciting results. Cancer stem cells (CSCs) attract the attention of researchers of ARTs because of their crucial role on tumor occurrence, metastasis and recurrence. Although only a few articles about how ARTs affect CSCs have been reported (Cao et al., 2014; Berte et al., 2016; Tong et al., 2016), these new discoveries might provide a revolutionary approach for cancer therapy.

In this review, recent progress for cancer therapy based on ARTs including the development of novel ARTs derivatives, novel nano-formulations and combination therapy is summarized and the latest findings of the mechanisms of action are analyzed for further understanding this huge potential in cancer treatment and promoting clinical application.

## Novel Design Strategies For Developing Anticancer Candidates Based On Arts

The antitumor activity of ARTs has attracted extensive attention (Firestone and Sundar, 2009; Crespo-Ortiz and Wei, 2012; Frohlich et al., 2016; Wong et al., 2017; Zhang Y. et al., 2018; Liu X. et al., 2019). To increase efficacy and reduce toxicity of ARTs, new design strategies for anticancer candidates based on ARTs have been developed. These strategies were raised by researchers from different fields. Medicinal chemists focus more on chemical structural modification to develop novel derivatives whereas pharmacists are more interested in formulation improvement. In our review, we elaborate these novel strategies by focusing on developing novel ART derivatives and manufacturing ART nano-formulations as well as combining ARTs with other drugs for cancer therapy.

### Developing Novel Derivatives With Enhanced Antitumor Activity

#### Mitochondria-Targeted ART Derivatives

**Figure 2:**
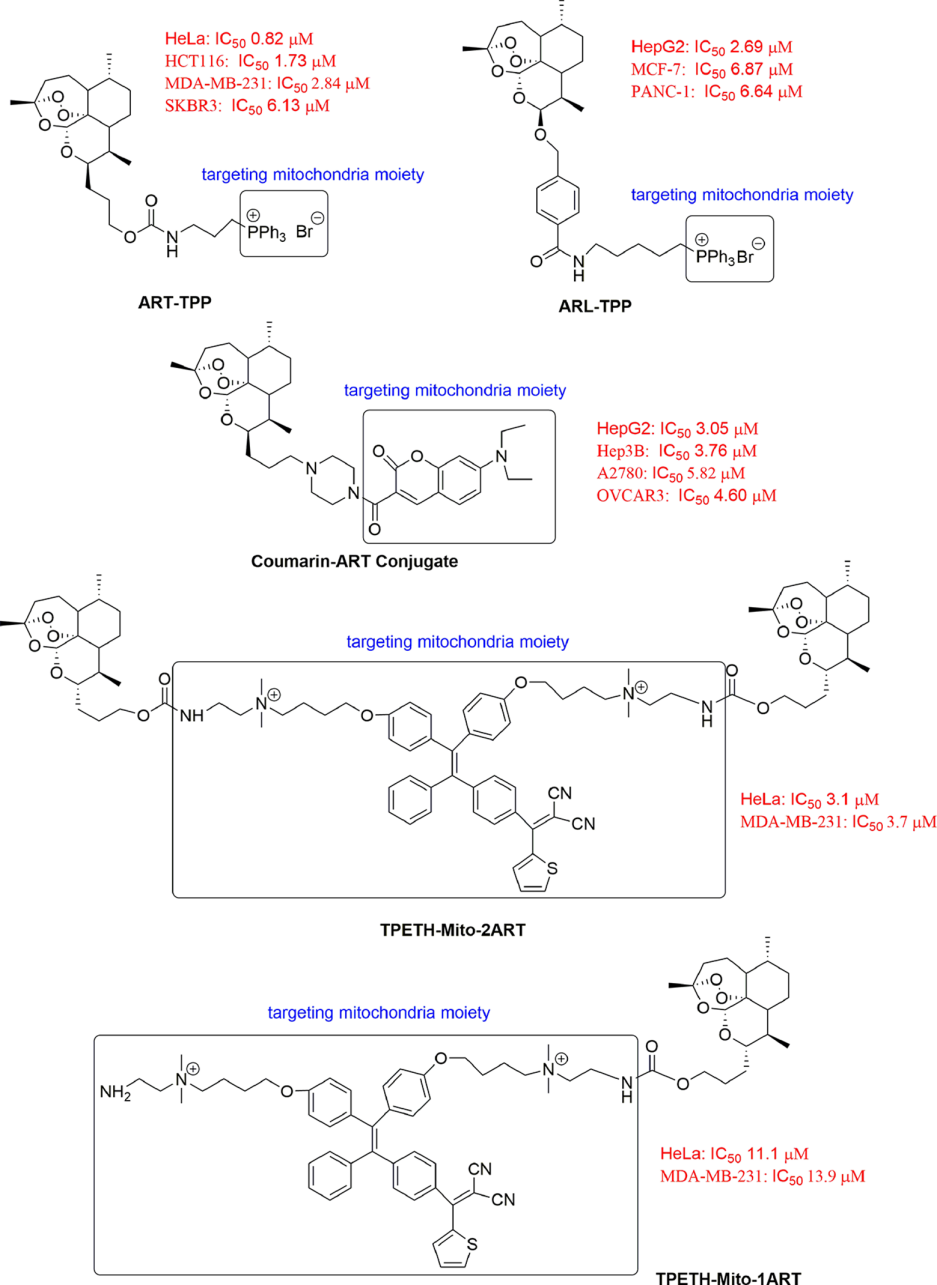
Novel mitochondria-targeted artemisinin derivatives.

Mitochondria are highly dynamic organelles involved in many cellular functions (Battogtokh et al., 2018). Mitochondria dysfunction has been observed in cancer cells (Gururaja Rao, 2017). Drugs specifically targeting mitochondria are therefore of therapeutic interest. Furthermore, current evidence of the mitochondrial membrane potential (Δψm) difference between normal and cancer cells has offered further confidence for designing drug to target mitochondria (Zielonka et al., 2017). The triphenylphosphonium cation (TPP^+^), one of delocalized lipophilic cations (DLCs) is used commonly to target parental compounds to the mitochondria (Ye et al., 2017; Jin et al., 2019). Mitochondria-targeted ART derivatives with enhanced antitumor activity have been synthesized as antitumor candidates (**Figure 2**). Zhang et al. synthesized a mitochondria-targeting ART analogue (ART-TPP) with enhanced anticancer activity to tested cancer cells, with the minimum IC_50_ value of 0.82 μM for HeLa cells and the maximum IC_50_ value of 6.13 μM for SKBR3 cells. The cytotoxicity of ART-TPP was more potent than ART alone in tested cells. The principle for the better activity of ART-TPP was confirmed by using a clickable probe. The probe localized well in the mitochondria after cellular uptake and bound to 209 proteins from mitochondria with more potential (Zhang et al., 2016). Sun et al. conjugated ARL to mitochondria-targeting TPP^+^ to obtain ARL-TPP. ARL-TPP significantly increased cytotoxic activity against MCF-7 cancer cells with an IC_50_ value of 6.87 μM, PANC-1 cancer cells with an IC_50_ value of 6.64 μM, HepG2 cancer cells with an IC_50_ value of 2.69 μM and LoVo cancer cells with an IC_50_ value of 2.73 μM (Sun et al., 2017). However, because TPP^+^ are nonemissive, Zhang et al. synthesized another novel class of fluorescent mitochondria-targeted coumarin–artemisinin conjugates to kill cancer cells by linking a mitochondrial dye, coumarin-3-carboximide with ART. These compounds had strong abilities to accumulate in mitochondria with enhanced anticancer activities, then the intracellular ROS levels increased efficiently and cell apoptosis was induced (Zhang X. et al., 2015). Feng et al. synthesized ART and aggregation-induced emission fluorogens (AIEgen) conjugates (TPETH-Mito-1ART and TPETH-Mito-2ART) for mitochondria-targeted and image-guided chemo- and photodynamic therapy for treating cancer. These conjugates largely improved cancer cell ablation efficacy with a synergistic effect by quickly depolarizing mitochondrial membrane and dramatically reducing cancer migration activity (Feng et al., 2018).

#### Hybrid Derivatives

Pharmacophore hybridization, a classical medicinal chemistry approach, involves combination of two or more functional groups *via* covalent bonds to produce a novel compound holding preferable biological activities (Meunier, 2008; Muregi and Ishih, 2010; Capci et al., 2019). This approach is often used in searching for safe, effective and specific innovative medicines for cancer therapy (Letis et al., 2017; Yu et al., 2018; Zborovskii et al., 2018). Many studies of ARTs have generated new hybrids, such as ART-derivative-N-heterocyclic carbene (NHC)-gold(I)-hybrids (Zhang et al., 2020), ART–daumone hybrids (Ma et al., 2018), DHA–bile acid hybrids (Letis et al., 2017; Marchesi et al., 2019), DHA–cinnamic acid hybrids (Xu et al., 2016), DHA–coumarin hybrids (Yu et al., 2018) and ART–chemotherapeutic agent conjugates (Li et al., 2016), et al., with more potent activity than with either agent alone (**Table 1**). We designed and synthesized DHA–cinnamic acid ester derivatives modified at two positions, C-9 and C-12. DHA-37 (namely, compound 17 in previous paper) was considered a candidate for treating lung cancer (Xu et al., 2016). Liu et al. found that DHA-37, different from DHA-induced apoptosis, increased the high mobility group box 1 (HMGB1) expression and induced autophagy by activating the MAPK signal but not PI3K-AKT-mTOR pathway (Liu et al., 2018). Zhang et al. synthesized ART-derivative-NHC-gold(I) hybrids which showed strong anticancer activities on a large panel of representative human cancer cell models with GI_50_ values in nanomolar (nM) range together with a high selectivity. This high selectivity might be due to inhibition of the redox antioxidant Nrf2 transcription factor. This transcript factor has been confirmed to be strongly associated with aggressiveness and resistance to cancer therapies (Zhang et al., 2020). Ma et al. found that ART–daumone hybrids inhibited cancer cell-mediated osteolysis by upregulating the level of activating transcription factor 3 and downregulating the level of E2F transcription factor 1 and hepatocyte nuclear factor 4 alpha (Ma et al., 2018). Li et al. found that ART-chemotherapeutic conjugates inhibited the growth and proliferation of ovarian cancer cells, resulting in S-phase arrest, apoptosis and epithelial mesenchymal transition (EMT) (Li et al., 2016). Furthermore, Yu et al. found that DHA-coumarin hybrids induced ferroptosis, promoted apoptosis, arrested the cell cycle progression, inhibited cell proliferation and migration (Yu et al., 2018).

**Table 1:** Summary of ARTs hybrids for antitumor activity.

Compound	Cell line	Event/Mechanism	References
ART-derivative-NHC gold(I) hybrids	A549, U-2 OS, MCF-7, T24, LAMA, HL-60, HepG2	Inhibit Nrf2 transcriptional activity	(Zhang et al., 2020)
ART-daumone hybrids	MDA-MB-231, A549	Tumor suppressive activating transcription factor 3↑, oncogenic E2F transcription factor 1↓, inhibit osteoclast formation, MMP 9↓, cathepsin K↓	(Ma et al., 2018)
DHA-bile acid hybrids	HL-60, HepG2,	Induce apoptosis	(Marchesi et al., 2019)
	CCRF-CEM, CEM/ADR5000	Enhance cytotoxicity, low cross resistance	(Letis et al., 2017)
DHA-cinnamic acid hybrids	A549	HMGB1↑, induce autophagy by activating MAPK signals	(Liu et al., 2018)
DHA-coumarin hybrids	HT-29, MDA-MB-231	Inhibit proliferation, arrest the cell cycle progression, induce both apoptosis and ferroptosis, inhibit migration	(Yu et al., 2018)
ART-chemotherapeutic agents conjugates	A2780, OVCAR-3	S-phase arrest, induce apoptosis and inhibit migration	(Li et al., 2016)
Thymoquinone-ART hybrids	CCRF-CEM, CEM/ADR5000	Better cytotoxicity than doxorubicin	(Fröhlich et al., 2018)
	HCT116, HT29	ROS↑, γ-H2AX↑	(Fröhlich et al., 2017)
ART-triazole hybrids	KB, HepG2	Exihibit moderate to good cytotoxic activities	(Tien et al., 2016)
ART-indole/imidazole hybrids	A549, MCF-7, HepG2, MDA-MB-231, MCF/ADR	Induce cell cycle arrest at G2 phase	(Hu et al., 2019)
ART-ferrocene hybrids	CCRF-CEM, CEM/ADR5000	Show a remarkable anticancer activity	(Reiter et al., 2015)
Tamoxifen/Estrogen-ART hybrids	PC-3, MCF-7	Enhance anticancer activity	(Fröhlich et al., 2020)

### Manufacturing Novel Nano-Formulations With Better Physicochemical Properties

Nano-formulations can achieve a drug-targeted distribution and increase the bioavailability of the drug to improve the curative efficacy, which is a key technology in targeted cancer therapy. To improve physicochemical properties of ARTs, researchers have developed ART-based nano-formulations such as liposomes (Gharib et al., 2014; Leto et al., 2016; Gao et al., 2018; Li H. et al., 2019; Liu J. J. et al., 2019), nanostructured lipid carriers (Emami et al., 2018), micelles (Nosrati et al., 2019; Wang Y. et al., 2019), nanospheres (Chen J. et al., 2015), nanocapsules (Meng et al., 2014; Tran et al., 2015) and multifunctional nanoparticles (Chen et al., 2014; Li et al., 2014; Wang et al., 2016a; Wang et al., 2016b; Pan et al., 2018) (**Table 2**). These nano-formulations overcame chemotherapeutic resistance with improved selectivity by targeting tumor cells. These novel nano-formulations according to the different design concepts and different delivery systems are summarized below.

**Table 2:** Summary of ART-based nano-formulations.

Nano-formations	Cell line	Event/mechanism	References
Liposomes	A549/R	Increase intracellular ROS generation and cell apoptosis rate	(Gao et al., 2018)
	MCF-7, MDA-MB-231	High antiproliferative activity in a magnetic field	(Gharib et al., 2014)
	HCT-8	Enhance anticancer activity	(Leto et al., 2016)
	HNSCC	Enhance anticancer activity by increasing DHA-targeted delivery and biocompatibility	(Li H. et al., 2019)
	NSCLC	VE-Cad↓, TGF-β1↓, MMP-2↓, HIF-1α↓, inhibit VM channels and tumor metastasis, increase the selective accumulation at tumor sites	(Liu J. J. et al., 2019)
Nanostructured lipid carriers	U87MG	Enhance anticancer activity	(Emami et al., 2018)
Micelles	MCF-7, MCF-7/ADR	Enhance antitumor activity and reduce toxicity	(Wang Y. et al., 2019)
	MCF-7, 4T1	High efficacy, low toxicity and tumor target	(Nosrati et al., 2019)
	CT-26	Enhance antitumor activity	(Hao et al., 2020)
Nanospheres	A549	Suppress tumor growth	(Chen J. et al., 2015)
Nanocapsules	L1210 and MCF-7	Decrease antitumor activity by controllable release of ART	(Meng et al., 2014)
	MCF-7, MDA-MB-231	Enhance anticancer activity	(Tran et al., 2015)
Multifunctional nanoparticles	HeLa	Enhance antitumor efficacy	(Chen et al., 2014)
	HeLa, A549	Significant cytotoxicity and no obvious side effects	(Wang et al., 2016a)
	HeLa	Synergy with combined chemo-photothermal therapy	(Wang et al., 2016b)
	HeLa, A375, HepG2	Enhance antiproliferative response compared with free drug	(Pan et al., 2018)
	C6, C6 stem cell	Destroy VM channels and induce apoptosis	(Li et al., 2014)

Based on a Fe^2+^/Fe^3+^-mediated Fenton reaction, magnetic DHA nanoliposomes were developed to circumvent cisplatin resistance and enhance targeted delivery and bioefficacy of DHA (Gao et al., 2018; Li H. et al., 2019). Based on transferrin overexpression in tumor cells, magnetic nanoliposomes and transferrin-conjugated liposomes were developed to target tumor *in vitro* and *in vivo* (Gharib et al., 2014; Leto et al., 2016). Also, ART-loaded transferrin-conjugated nanostructured lipid carriers were developed to increase water solubility, site specificity, selective targeting, efficient penetration, glioma cell distribution and internalization, as well as effective delivery across the blood–brain barrier with much lower drug concentration, greater therapeutic effect and decreased likelihood of neurotoxicity (Emami et al., 2018).

On the basis of pH-responsive degradation, novel Fe_3_O_4_@C@MIL-100(Fe) nanoparticles (Wang et al., 2016a), dual metal-organic-frameworks nanoparticles (Wang et al., 2016b), lipid nanoparticles (Zhang Y. J. et al., 2015), multifunctional nanocarriers (Chen et al., 2014), nanospheres (Chen J. et al., 2015), fluorescent magnet theranostic nanoparticles (Pan et al., 2018) and polymeric micelles (Hao et al., 2020) loading ARTs were fabricated. These nano-formulations possessed pH-responsive property since they relied on acidified tumor microenvironment along with a further acidified endosome/lysosome network. Once these nano-formulations were endocytosized into tumor, they controllably released incorporated drugs to take effect, or Fe^2+^ ions or Mn^2+^ ions, which converted ARTs to highly active products to enhance cell killing.

In addition, arginine _8_ modified DHA-epirubicin liposomes were developed with ideal physicochemical characteristics for powerful cytotoxicity against A549 cells and these liposomes effectively suppressed vasculogenic mimicry (VM) channels and blocked tumor metastasis (Liu J. J. et al., 2019). mPEG–ART nanocapsules were synthesized based on a mPEGylated ARS pro-drug but conferred decreased cytotoxicity than free ARS (Meng et al., 2014). Fortunately, ARS-loaded chitosan-coated lipid nanocapsules were developed with stronger antitumor activity than free ART (Tran et al., 2015). Doxorubicin and DHA co-loaded Soluplus^®^-TPGS and biotin-functionalized copolymeric PEG-PCL micelles were developed with higher antitumor activity and lower toxicity (Nosrati et al., 2019; Wang Y. et al., 2019). These newly developed nano-formulations provided novel therapeutic candidates with high efficiency for treating cancer.

### Combination Therapy

Combination therapy approach can be used to increase efficacy, reduce toxicity and overcome drug resistance (Suraweera et al., 2018). Resistance to ARTs has already appeared in malaria treatment. New partner drugs of ARTs have been suggested to establish combination-treatment regimen for antimalaria to reduce resistance risk (Wang J. et al., 2019). Successful combinations of ARTs with chemotherapeutics or phytochemicals have been highlighted in recent years (**Table 3**). These findings provided promising therapeutic approaches for cancer therapy.

**Table 3:** Summary of ARTs combinations with chemotherapy drugs and phytochemicals.

Compound	Cell lines	Event/mechanism	References
DHA-cisplatin	SKOV3/DDP	mTOR inhibition, promote apoptosis	(Feng et al., 2014)
	A549, A549/DDP	Suppress the expression of HIF-1α and VEGF to effect tumor angiogenesis, increase apoptosis	(Zhang et al., 2013)
	A549, LLC	Inhibit tumor growth and metastasis	(Zhou et al., 2010)
ARS-cisplatin	A2780, HO8910	Downregulate RAD51	(Wang et al., 2015)
DHA-carboplatin	A2780, OVCAR-3	Death receptor- and mitochondrion-mediated caspase-dependent apoptotic pathways	(Chen et al., 2009)
	LLC	Inhibit cell proliferation, induce G0/G1 phase cell cycle arrest, increase cell apoptosis, p38 MAPK activation	(Zhang B. et al., 2018)
DHA-5-FU	HCT116 TP53^-/-^	Inhibit proliferation, induce ROS-mediated apoptosis and Bcl-2/Bax↓	(Yao et al., 2018)
DHA-gemcitabine	NCI-H1975	G2/M phase cell cycle arrest, cyclin B1↓, cyclin-dependent kinase 1↓, inhibit the migratory and invasive, promote apoptosis p-Akt↓ p-mTOR↓ p-STAT3↓ Bcl-2↓ Bax↑, Akt/mTOR/STAT3 pathway	(Hong et al., 2017)
	A549	Induce apoptosis through bak-mediated intrinsic pathway and fas-caspase-8-mediated extrinsic pathway	(Zhao et al., 2014)
	BxPC-3, PANC-1	Inactivate NF-κB	(Wang et al., 2010)
ARS-cytarabine, DHA-cytarabine	human AML cell lines	Produce initial regression, but did not prolong survival *in vivo*	(Drenberg et al., 2016)
DHA-ABT-263	BCR-ABL^+^B-ALL leukemic cells	Down-regulate MCL-1 expression	(Budhraja et al., 2017)
	Non-small cell lung cancer	Inhibit STAT3 activity, modulate the expression of MCL-1, Survivin and Bim	(Yan et al., 2018)
ARS-erlotinib	glioblastoma multiforme cell lines	Activate EGFR	(Efferth et al., 2004; Efferth, 2017a)
ARS-sorafenib	Huh7, SNU-449, and SNU-182 HCC cell lines	Induce ferroptosis	(Li et al., 2020)
DHA-curcumin	SKOV3	Decrease cell viability, arrest cell cycle, promote apoptosis, MK↑, miR-124↑	(Zhao et al., 2017)
	lethal(2)giant larvae, [l(2)gl] brain tumor	Prolong life span, restore locomotor activity	(Das et al., 2014)
ARS-triptolide	PANC-1, CFPAC-1	Inhibit cell growth, induce apoptosis, HSP 20↓, HSP 27↓	(Liu and Cui, 2013)
DHA-dictamnine	A549	Induce caspase-3–dependent apoptosis	(An et al., 2013)
DHA-dexamethasone	multiple myeloma cells	ROS↑, △Ψm↓, induce caspase-medicated apoptosis, overcome resistance	(Chen Y. et al., 2020)
ARS-bicalutamide	PC3, 22RV1, LNCaP	Inhibit NF-κB signaling and decreases AR and/or AR-variant 7 expression *via* ubiquitin-mediated proteasomal degradation, induce oxidative stress and apoptosis *via* survivin downregulation and caspase-3 activation, resulting in poly-ADP-ribose polymerase cleavage	(Nunes et al., 2017)
DHA-doxorubicin	mutant p53 (R248Q)-expressing Hep3B	Decrease P-gp expression *via* inhibiting the p53 (R248Q)-ERK1/2-NF-κB signaling pathway	(Yang et al., 2019)

Multiple mechanisms were found in these combinations. The *in vivo* and *in vitro* applications of DHA–cisplatin combinations were mainly mediated by inhibiting mTOR (Feng et al., 2014), reducing tumor microvessel density (Zhang et al., 2013) and inhibiting tumor growth and metastasis (Zhou et al., 2010). However, ARS–cisplatin combination was mediated by downregulating RAD51 (Wang et al., 2015). DHA–carboplatin combination was mediated by the mitochondrion and death receptor-mediated caspase-dependent apoptotic pathway (Chen et al., 2009) and p38 mitogen-activated protein kinase (MAPK) activation (Zhang B. et al., 2018). Combinations with the antimetabolite 5-fluorouracil involved ROS (Yao et al., 2018), whereas that with gemcitabine was regulated by the Fas-caspase-8–mediated extrinsic pathway and Bak-mediated intrinsic pathway (Zhao et al., 2014), deactivating gemcitabine-induced NF-kB activation (Wang et al., 2010) or promoting apoptosis *via* the Akt/mTOR/signal transducer and activator of transcription 3 (STAT3) pathway (Hong et al., 2017). *In vivo*, Combinations of ARS or DHA with cytarabine produced initial regression but did not prolong survival (Drenberg et al., 2016). Of note, synergy between kinase inhibitors and DHA or ARS involve different mechanisms from those mentioned above, including repressing myeloid cell leukemia 1 (MCL-1) expression (Budhraja et al., 2017; Yan et al., 2018), inhibiting STAT3 activity (Jin et al., 2017; Yan et al., 2018), activating epidermal growth factor receptor (EGFR) (Efferth et al., 2004; Efferth, 2017a) and inducing ferroptosis (Li et al., 2020). DHA–curcumin combination decreased the expression of oncogene midkine and upregulated the expression of the microRNA miR-124 to induce apoptosis (Zhao et al., 2017). However, the ART–curcumin combination prolonged the life span and restored locomotor activity *via* ROS mediated in *Drosophila* in brain tumor (Das et al., 2014). In addition, ARS-triptolide combination inhibited pancreatic cancer cell line growth and induced apoptosis, accompanying downregulation of the expression of heat shock proteins (HSP) 20 and HSP 27 (Liu and Cui, 2013). DHA-dictamnine combination dramatically increased apoptotic cell death *via* a caspase dependent pathway in human lung adenocarcinoma cells (An et al., 2013).

Overcoming drug resistance using combinations of ARTs with other drugs is another advantage for cancer therapy. Chen et al. reported that DHA treatment overcame dexamethasone resistance and enhanced dexamethasone efficacy in multiple myeloma by increasing ROS production and cytochrome C translocation from mitochondria to cytoplasm, resulting in alterations to Δψm and caspase-mediated apoptosis (Chen Y. et al., 2020). Yao et al. reported that DHA effectively restored anticancer activity of 5-FU against 5-FU resistant HCT116 TP53^-/-^ cells through ROS-mediated apoptosis and upregulation of the bcl-2/bax expression ratio (Yao et al., 2018). Nunes et al. reported that combination of ARS and bicalutamide restored sensitivity of castrate-resistant prostate cancer cells to antiandrogens. The mechanisms involved in inhibiting NF-κB signaling, together with decreased expression of androgen receptor (AR) and/or AR-variant 7 and the induction of oxidative stress and apoptosis (Nunes et al., 2017). Mutant p53 (R248Q) can induce doxorubicin resistance in hepatocellular carcinoma (HCC). Yang et al. reported that DHA sensitized mutant p53 (R248Q)-expressing HCC to doxorubicin. The mechanism of action involved in reducing P-gp expression *via* inhibiting the p53 (R248Q)-ERK1/2-NF-κB signaling pathway (Yang et al., 2019). It is well known that treatment of arsenic trioxide to lung cancer cells easily developed high level of resistance (Chen H. et al., 2017). Chen et al. reported that combination of DHA and arsenic trioxide reduced arsenic trioxide resistance of lung cancer cells by increasing cellular level of ROS and DNA damage. Moreover, treatment of normal human cells with the combination did not result in significant adverse events (Chen H. et al., 2017).

In conclusion, these novel combination approaches based on ARTs improved the antitumor activity and led to the development of new drug partners for clinical application. Furthermore, several newly developed candidates also presented new mechanisms of action including in autophagy (Liu et al., 2018), in ferroptosis (Yu et al., 2018) and in Nrf2 signaling (Zhang et al., 2020), which warrant to be noted.

## The Effects of Arts on Cell Signaling Pathways and Modes of Induction of Cell Death

**Figure 3:**
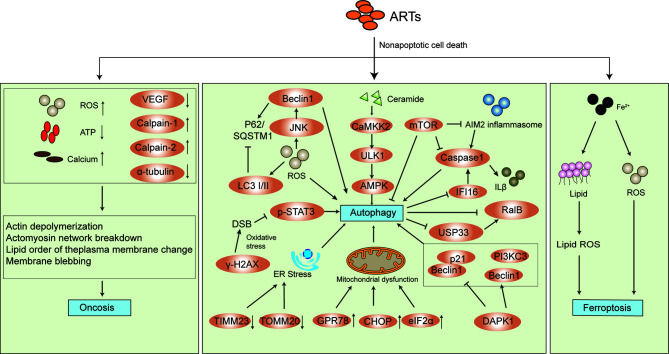
New mechanisms of action of ARTs-mediated non-apoptotic cell death.

The antitumor activity of ARTs involves multitargets and multipathways as mentioned above. Inducing cell cycle arrest is one of some common ones to inhibit proliferation of tumor cells (Jia et al., 2016; Chen J. et al., 2019), inhibit tumor cell invasion and metastasis (Rasheed et al., 2010; Weifeng et al., 2011), exerting antiangiogenic effects against tumor cells (Wei and Liu, 2017) and inducing cancer cell apoptosis (Jia et al., 2016; Chen J. et al., 2019). More recently, several new mechanisms on non-apoptotic cell death e.g. oncosis, autophagy and ferroptosis have been proposed. As shown in **Figure 3**, the mechanisms of action involve multiple cell signaling pathways. Besides, reports showed that ARTs regulated cancer cell metabolism including glucose metabolism and lipid metabolism, inhibited immunosuppression and the stemness of cancer stem cells in various cancer cell lines (Chen X. et al., 2017; Cao et al., 2019; Chen X. et al., 2019; Chen S. et al., 2020; Gao et al., 2020). These new findings further highlight the multitarget bioactive properties and complexity of the ART-mediated anticancer effect.

### The Effects of ARTs on Non-Apoptotic Cell Death

#### Oncosis

Oncosis is characterized by rapid cell swelling, organelle swelling, membrane permeability, and cell lysis. It is associated with intercellular events including increased ROS generation, mitochondrial swelling, adenosine triphosphate (ATP) depletion, failure of Ca^2+^ homeostasis, activation of certain proteases (e.g., calpains and cathepsins), lysosomal disruption, and eventually plasma membrane rupture (Majno and Joris, 1995; Van Cruchten and Van Den Broeck, 2002; Golstein and Kroemer, 2007; D’arcy, 2019). It has been shown that ARS induced oncosis-like cell death in pancreatic cancer and renal cell carcinoma (Du et al., 2010; Jeong et al., 2015). The former cell death occurred with the morphotype characteristics of oncosis and the latter *via* ROS generation and ATP depletion (Du et al., 2010; Jeong et al., 2015). Interestingly, in gastric cancer, ARS promoted cell oncosis by decreasing the expression of VEGF and increasing the amount of calcium and the expression of calpain-2 (Zhou et al., 2013).

#### Autophagy

Autophagy consists of the degradation and recycling of organelles and portions of the cytosol (Parzych and Klionsky, 2014; Saha et al., 2018). As a “self-eating” process and well-known type II programmed cell death, autophagy acts as a double-edged sword because of its association with both cell survival and death. It has been verified that ARTs induced autophagy (Du et al., 2013; Feng et al., 2014; Jia et al., 2014; Qu et al., 2017; Shi et al., 2017; Wang et al., 2017; Cheng et al., 2018; Jiang et al., 2018; Thongchot et al., 2018; Guan and Guan, 2020). However, how autophagy accelerates cell death and enhances the cell survival after ARTs exposure are contradictory. Some evidence showed that autophagy induced by ARS and DHA was to protect cancer cells (Jia et al., 2014; Jiang et al., 2018), whereas autophagy induced by DHA was to kill cancer cells (Qu et al., 2017). Other evidence of autophagy-depended ferroptosis, cell cycle arrest and cell apoptosis induced by ARTs and ARTs-induced autophagy sensitized chemotherapy drugs to enhance the cell death demonstrated the killing effect (Feng et al., 2014; Zhang Z. S. et al., 2015; Cheng et al., 2018; Du et al., 2019; Ma et al., 2020), whereas autophagy inhibitor enhanced the anticancer property of ARTs, indicating the protecting effect of autophagy (Chen S. S. et al., 2015).

The molecular mechanisms of ART-induced autophagy involved accumulation of ROS, which activated JNK pathway in pancreatic cancer cells (Jia et al., 2014), or stimulating *de novo* synthesis of ceramide and CaMKK2–AMPK–ULK1 axis, which in turn cause the occurrence of autophagy in diffuse large B-cell lymphomas (Cheng et al., 2018), or increasing the expression of death-associated protein kinase 1 (DAPK1), reducing the interaction of beclin1 with bcl-2 and promoting the interaction of beclin1 with PI3KC3 in cholangiocarcinoma (Thongchot et al., 2018). Alternatively, it might cause endoplasmic reticulum stress and mitochondrial dysfunction in human glioblastoma cells (Qu et al., 2017). ARTs-induced autophagy was also mediated by inhibiting the nuclear localization of phosphorylated STAT3 in human tongue squamous cell carcinoma cells (Shi et al., 2017), as well as inhibiting the Akt/mTOR signaling pathway in esophageal cancer cells (Chen X. et al., 2020). Furthermore, ARTs regulated the crosstalk between autophagy and inflammasomes, which induced the activation absent in melanoma 2 (AIM2)/caspase-1 inflammasome to trigger autophagy in hepatocellular carcinoma and induced autophagy with suppressing the activation of IFI16/caspase-1 inflammasome and IL-1β production together with reducing the expression of ubiquitin-specific processing protease 33 (USP33) and Ras like B (RalB) in human laryngeal squamous cell carcinoma (Shi et al., 2019; Shi et al., 2020).

#### Ferroptosis

Ferroptosis, unlike traditional apoptosis and necrosis, is a novel type of caspase-independent non-apoptotic cell death. It is caused by accumulation of iron-dependent lipid peroxide and is characterized mainly by cell volume shrinkage and enhanced mitochondrial membrane density without typical apoptotic and necrotic manifestations (Dixon et al., 2012; Xie et al., 2016; Lu et al., 2017). ARTs exhibited anticancer activity related to ferric iron and ROS which is similar to ferroptosis. Ooko et al. found that the expression of numerous iron-related genes including genes encoding transferrin, transferrin receptors 1 and 2, cerulopasmin and lactoferrin were significantly correlated to the log_10_IC_50_ values for ARTs, indicating ferroptosis-inducing activity of ARTs (Ooko et al., 2015). Subsequently, ARTs-induced ferroptosis was found in various types of cancer cells *in vitro* and *in vivo* (Lin et al., 2016; Roh et al., 2017; Du et al., 2019; Wang N. et al., 2019; Chen G. Q. et al., 2020). The underlying molecular mechanisms involved the decrease of the protein levels of glutathione peroxidase 4 (GPX4) and rat sarcoma (Ras) in head and neck squamous cell carcinoma cells (Lin et al., 2016), activation of the Nrf2–antioxidant response element pathway in head and neck cancer cells (Roh et al., 2017) and the ATF4-CHOP-CHAC1 pathway in Burkitt’s lymphoma (Wang N. et al., 2019). Recently, Chen and Wang et al. demonstrated that heat shock protein family A member 5 (HSPA5), also termed GRP78, is a negative regulator of DHA or ARS-induced ferroptosis in *KRAS* mutant pancreatic cancer cells and glioma cells (Chen Y. et al., 2019; Wang K. et al., 2019).

### The Effects of ARTs on Cancer Metabolism

Metabolic changes have been demonstrated in cancer cells compared to normal non-malignant cells. Warburg effect describes a phenomenon in which, despite the presence of oxygen, cancer cells preferentially metabolize glucose by glycolysis to produce lactate as an end product (Warburg, 1956; Hsu and Sabatini, 2008). ARTs inhibited the glycolysis capacity in various tumor cells. Mi et al. first reported that DHA suppressed glucose uptake and glycolysis in non-small-cell lung carcinoma cells and confirmed the effect associated with inhibiting mTOR activity and reducing glucose transporter 1 (GLUT1) expression (Mi et al., 2015). Subsequently, Vatsveen et al. observed that ARS decreased glycolysis capacity and mitochondrial respiration capacity in B-cell lymphoma cells although detailed mechanisms remain to be elucidated (Vatsveen et al., 2018). However, inhibition of glycolysis of DHA was illustrated *via* inhibiting PI3K/AKT pathway, downregulating HIF-1α expression and down-regulating pyruvate kinase M2 (Li S. et al., 2019; Zhu et al., 2019; Gao et al., 2020). The oxidative pentose phosphate pathway, another catabolic pathway of glucose, is important for tumor growth and cancer cell metabolism. Elf et al. (2017) reported that targeting 6-phosphogluconate dehydrogenase could sensitize leukemia cells to DHA in oxidative pentose phosphate pathway. Besides an effect on glycolytic metabolism, ARS inhibited HCT116 colon cancer cell proliferation by suppressing the fatty acid biosynthetic pathway, mainly downregulating three proteins: acyl-CoA synthetase 5, hydroxyacyl-coenzyme A dehydrogenase and fatty acid synthase (Chen X. et al., 2017).

### The Effects of ARTs on Immunosuppression

The immunosuppressive properties of ARTs in tumor cells have been reported. ART hampered 4T1 tumor growth by promoting T cell activation and quelling immunosuppression from Tregs and MDSCs (Cao et al., 2019). After ART treatment, MDSCs and Tregs frequencies were significantly decreased and those of D4^+^ interferon γ^+^ T cells and cytotoxic T lymphocytes were significantly increased. The mRNA levels of T-bet, interferon γ, and tumor necrosis factor α were significantly increased and the mRNA level of transforming growth factor β (TGF-β) was significantly decreased. However, the expressions of interleukin 10 (IL-10) and forkhead box P3 (Foxp3) did not change significantly, inconsistent with previous reports (Zhang et al., 2014; Cui et al., 2015). The level of IL-10 was decreased greatly in colorectal cancer and that of Foxp3 decreased in cervical cancer after ARS treatment. In colorectal cancer, ARS downregulated the immunosuppression by decreasing TGF-β1 and IL-10 levels (Cui et al., 2015), which will be beneficial for colorectal tumor patients with higher TGF-β1 and IL-10. In cervical cancer, ARS inhibited prostaglandin production, which in turn led to the decreased expression of Foxp3 in T cells (Zhang et al., 2014). In addition, the expression of miRNAs was affected by ARS treatment, leading to regulation of immunosuppression. In ovarian cancer, ARS up-regulated miR-142 that in turn suppressed Sirt1 level and promoted T helper 1 cell differentiation, thereby enhancing cell apoptosis (Chen X. et al., 2019). In bladder cancer, ARS up-regulated miR-16 expression, which decreased cyclooxygenase 2 expression and prostaglandin production (Jiang et al., 2018).

### The Effects of ARTs on Cancer Stem Cells

Cancer stem cells (CSCs) have stem-like properties, with a unique ability for self-renewal, proliferation and differentiation. They play a crucial role in tumor occurrence, metastasis and recurrence (Reya et al., 2001). Recent studies showed that ARTs inhibited the expression of the CSC markers Nanog, Oct3/4, ALDH1, CD44 and Sry-related high mobility group box (SOX2) and cell sphere formation ability (Tong et al., 2016; Chen S. et al., 2020), exhibited anti-CSC proliferation (Cao et al., 2014) and induced CSC apoptosis (Li et al., 2014). They even synergistically enhance anti-CSC proliferation of chemotherapeutic drugs (Berte et al., 2016). Mechanisms included inhibiting p-AKT and activating caspase-3, disturbing mitochondrial metabolism and down-regulating the expression of RAD51, an important component of DNA double-strand break repair (Cao et al., 2014; Berte et al., 2016; Subedi et al., 2016). PI3K/Akt, MEK/ERK and Wnt/β-catenin signaling pathways were involved in the inhibition of cancer cells stemness of ARTs (Tong et al., 2016; Chen S. et al., 2020). These new findings imply that ARTs can use as CSCs inhibitor for cancer therapy.

## Clinical Trials

We searched for clinical trials of ARTs at ClinicalTrials.gov. and in PubMed. ClinicalTrials.gov search results by keywords “cancer” and “artesunate” show that some clinical trials of ARS have entered phase II status in recent years. Different modes of administration are used for different cancers. However, no solid final results can be seen yet. In Pubmed, there are only 3 studies in the last 3 years. We summarize the latest research results in 2019 because some previous clinical studies in dogs and humans have been comprehensively reviewed by Efferth and Zhang et al. (Efferth, 2017b; Zhang Y. et al., 2018).

Recent clinical studies for treating cancer focus on ARS. The results showed dose-limiting toxicities could be seen at dosages of 12, 18 and 25 mg/kg by intravenous ARS in patients with advanced solid tumor malignancies and the maximum tolerated dose was 18 mg/kg using day1/day8, 3-week cycle of administration (Deeken et al., 2018). The adverse events of auditory and vestibular system in patients with metastatic or locally advanced breast cancer after 4 weeks of add-on therapy with oral ARS possibly related to the intake of ARS. However, none of audiological results showed any dose-limiting auditory toxicity and adverse events was fully reversible after discontinuation of ARS, indicating that audiological monitoring in further clinical studies with prolonged use of oral ARS in doses up to 200 mg daily is necessary (Konig et al., 2016). Subsequently, a short-term dose-finding study in metastatic breast cancer patients also showed a well-tolerated dose of oral ARS was 200 mg (2.2–3.9 mg/kg/d). Three patients experienced leucopenia, neutropenia, asthenia, anemia, dose-limiting adverse events altogether, possibly related to ARS (Von Hagens et al., 2017). However, short term trials only reveal limited safety information, rarely generally needed for long-term treatment in advanced cancer. Von Hagens et al. reported a long term add-on therapy (compassionate use) study with oral ARS in metastatic breast cancer after participating in a phase I study (ARTIC M33/2) to ensure adequate individual safety and tolerability (Von Hagens et al., 2019). 13 patients continued the add-on therapy as compassionate use. A total of 25 adverse events grade ≥ 2 at least possibly related to ARS long-term add-on therapy were documented, two, six and seventeen in dose groups 100, 150 and 200 mg/d ARS respectively. Six of these adverse events were classified as grade 3, two in patients taking 150 and four in patients on 200 mg/d, indicating the dose-dependent toxicity. However, none of them was probably or certainly related to ARS.

In summary, ARS was well tolerated and safe in patients with solid tumor malignancies (Deeken et al., 2018) and metastatic breast cancer (Konig et al., 2016; Von Hagens et al., 2017; Von Hagens et al., 2019). However, safety monitoring by reminders on ARS administration to detect the occurrence of side-effects must be considered especially when ARS is used in high dose. If necessary, drugs to prevent side-effects should be combined. Moreover, although it is difficult to differentiate between disease-related and drug-induced adverse events in cancer patients, current results show that adverse events were possibly related to the intake of ARS. Whether adverse events are certainly induced by ARS should be determined in future clinical trials. Some factors e.g. mode of administration, dosage and internal and duration of drug administration influence the safety and efficacy. A detailed examination of these factors needs to be taken seriously in future. Finally, currently available phase I clinical trial results of ARTs for treating cancer are still largely limited and the number of participating patients is small. In future, large scale phase II, III and IV clinical trials are needed to provide more convincing evidence for the suitability of ARS in clinical oncology, and more clinical trials of other ARTs such as DHA, ARM and ARE etc. should be advanced.

## Summary and Outlook

ARTs which are already established as safe drugs for treating malaria, possess a host of advantages that make them worthy of development as novel anticancer agents. They differ from available anticancer drugs because of the characteristics of high selectivity and efficacity against multiple cancers in cell and biological models as well as more sensitivity to chemoradiotherapy and less susceptibility to resistance. In this review, we summarized novel cancer therapeutic approaches based on ARTs, the latest molecular mechanisms of action and clinical studies. As a whole, ARTs have great potential to be used in clinical oncology, but there are still many problems to be solved.

First, because of poor solubility, short half-life, low bioavailability and toxicity of ARTs, developing novel derivatives and nanodrugs were applied to solve these problems. However, despite the cost savings in drug development by structural modification of ARTs to obtain candidates, updated preclinical and clinical studies of these modified derivatives are still limited. Besides, the aim of developing nanodrugs is to improve the physicochemical property of ARTs, but subsequent pharmacokinetic studies of these nanodrugs are rarely reported. Whether these new preparations actually improve the pharmacokinetic parameters needs to be further clarified.

Second, though the promising anticancer activity of ARTs has been identified *in vitro* and *in vivo*, the dose used is at μM level, still high in comparison with nM level of antimalarial activity. The higher the dose, the greater the possible side effects. Dose-dependent neurotoxicity has become the biggest obstacle to develop ARTs as clinical drugs (Genovese et al., 2000; Li et al., 2005). The toxicity to neuron should be considered when screening drugs. Furthermore, monitoring side-effect, especially neurotoxicity must be performed in future to successfully obtain promising drug candidates.

Third, the accurate mechanism of action is still controversial, and the target of action has not been completely elucidated. Current studies show that ARTs can affect tumor progression by multi-approaches and multi-links. Apoptotic cell death and non-apoptotic cell death are both involved in the antitumor activity of ARTs. In addition, ARTs have effects on cancer metabolism, immunosuppression and CSCs. However, the related literature is still relatively scarce. More in-depth research into these aspects is needed.

Finally, clinical study of cancer patients needs to be advanced for not only first-generation derivatives but also newly developed derivatives to obtain more comprehensive information of ARTs. The eventual development of such derivatives into approved drugs for cancer chemotherapy will be enormously important.

## Author Contributions

CX and XY conceived, designed, drafted the manuscript, and amended the paper. HZ designed the figures and tables. LM collected the related research articles. All authors contributed to the article and approved the submitted version.

## Funding

This work was supported by the Scientific Research Project of Education Department in Hunan Province (17C0973) to CX; and the National Natural Science Foundation of China (81874212); the Key Laboratory of Study and Discovery of Small Targeted Molecules of Hunan Province, Hunan Normal University (2017TP1020); and Huxiang High-Level Talent Innovation Team (2018RS3072) to XY.

## Conflict of Interest

The authors declare that the research was conducted in the absence of any commercial or financial relationships that could be construed as a potential conflict of interest.

## Abbreviations

Akt, protein kinase B; Bak, Bcl-2 homologous antagonist/killer; Bcl-2, B cell lymphoma-2; Bax, Bcl-2–associated X protein; PI3K, phosphatidylinositol 3-kinase; mTOR, mammalian target of rapamycin; LC3, light chain 3; CaMKK2, Ca^2+^/calmodulin-dependent kinase kinase 2; ULK1, unc-51 like autophagy activating kinase 1; PI3KC3, class III phosphatidylinositol 3-kinase; T-bet, T-box expressed in T cells; MEK, MAPK - mitogen activated protein kinase; ERK, extracellular signal-regulated kinase; Nrf-2, nuclear factor (erythroid‐derived 2)‐like 2; VEGF, vascular endothelial growth factor; JNK, c-Jun-N-terminal kinase; γ-H2AX, phosphorylated histone H2AX; DSB, double-strand break; IFI 16, Interferon-inducible 16; IL, interleukin; ATF4, activating transcription factor 4; CHOP, C/EBP homologous protein; CHAC1, cation transport regulator-like protein 1; ALDH1, aldehyde dehydrogenase-1.
